# Neoepitope load, T cell signatures and PD-L2 as combined biomarker strategy for response to checkpoint inhibition immunotherapy

**DOI:** 10.3389/fgene.2023.1058605

**Published:** 2023-03-23

**Authors:** Annie Borch, Anne-Mette Bjerregaard, Vinicius Araujo Barbosa de Lima, Olga Østrup, Christina Westmose Yde, Aron Charles Eklund, Morten Mau-Sørensen, Carolina Barra, Inge Marie Svane, Finn Cilius Nielsen, Samuel A. Funt, Ulrik Lassen, Sine Reker Hadrup

**Affiliations:** ^1^ Department of Health Technology, Technical University of Denmark, Lyngby, Denmark; ^2^ Department of Bioinformatics and Datamining, Novo Nordisk, Bagsvaerd, Denmark; ^3^ Department of Oncology, Phase 1 Unit, Rigshospitalet, Copenhagen, Denmark; ^4^ Center for Genomic Medicine, Rigshospitalet, Copenhagen, Denmark; ^5^ Clinical Microbiomics A/S, Copenhagen, Denmark; ^6^ Department of Health Technology, Section for Bioinformatics, Technical University of Denmark, Lyngby, Denmark; ^7^ National Center for Cancer Immune Therapy, Copenhagen University Hospital, Herlev, Denmark; ^8^ Department of Clinical Medicine, University of Copenhagen, Copenhagen, Denmark; ^9^ Weill Cornell Medical College, New York, NY, United States

**Keywords:** tumor mutational burden, neoepitopes, T cell signatures, immunotherapy, immune checkpoint inhibition, biomarker, programmed cell death 1 ligand 2

## Abstract

Immune checkpoint inhibition for the treatment of cancer has provided a breakthrough in oncology, and several new checkpoint inhibition pathways are currently being investigated regarding their potential to provide additional clinical benefit. However, only a fraction of patients respond to such treatment modalities, and there is an urgent need to identify biomarkers to rationally select patients that will benefit from treatment. In this study, we explore different tumor associated characteristics for their association with favorable clinical outcome in a diverse cohort of cancer patients treated with checkpoint inhibitors. We studied 29 patients in a basket trial comprising 12 different tumor types, treated with 10 different checkpoint inhibition regimens. Our analysis revealed that even across this diverse cohort, patients achieving clinical benefit had significantly higher neoepitope load, higher expression of T cell signatures, and higher PD-L2 expression, which also correlated with improved progression-free and overall survival. Importantly, the combination of biomarkers serves as a better predictor than each of the biomarkers alone. Basket trials are frequently used in modern immunotherapy trial design, and here we identify a set of biomarkers of potential relevance across multiple cancer types, allowing for the selection of patients that most likely will benefit from immune checkpoint inhibition.

## Introduction

Immune checkpoint inhibition (ICI) is an approved treatment modality for several cancer types, and various novel combinations of ICI are being tested in a large number of clinical trials (Tang et al., 2018). Despite the success of this treatment modality, a substantial fraction of patients does not respond. Consequently, there is an urgent need to identify biomarkers that allow for the selection of patients that are most likely to benefit from ICI. Tumor mutational burden (TMB), defined as the number of non-synonymous mutations, has been demonstrated as a potential biomarker (Chan et al., 2018) also across a diverse set of cancers (Goodman et al., 2017). However, it is evident that TMB as a single parameter does not apply to all patient groups (Blank et al., 2018; Holm et al., 2022). The TMB is believed to drive the antigen recognition of tumors, and hence the predicted neoepitope load might serve as an even better parameter to determine the tumor immunogenicity. Neoepitope load consists of the number of predicted neopeptides originating from non-synonymous mutations, potentially presented by the human leucocyte antigen (HLA) class I molecules. Studies show that the clinical benefit of immunotherapy is associated with high neoepitope load across multiple cancer types (Wells et al., 2020), underlining the possibility of using this parameter as a biomarker, but not all studies agree with this assertion (Wood et al., 2020). Programmed cell death protein 1 (PD-1), Programmed death ligand 1 (PD-L1), and 2 (PD-L2) have been discovered as single biomarkers for ICI treatments that block the PD-L1/PD-L2 to PD-1 interaction, but the predictiveness of these biomarkers are complex and do not work for all patients (Latchman et al., 2001; Yearley et al., 2017; Yang et al., 2019; Burdett and Desai, 2020).

Evaluation of novel immunotherapeutic treatments for cancer is frequently initiated using a “basket trial” design, as most of such therapies can potentially benefit patients with different cancer diagnoses, and that features of responsiveness often appear to be similar across different cancer indications. This indeed holds true for both expression of PD-L1 (Herbst et al., 2014; Doroshow et al., 2021), T cell infiltration (Ros-Martínez et al., 2020), and TMB (Samstein et al., 2019; Sha et al., 2020). In the present study, we evaluate a diverse patient group for potential genetic signatures that can be relevant for response to ICI. We utilize whole exome sequencing (WES), RNA sequencing (RNAseq), and expression arrays from patients treated with ICI to investigate the impact of high TMB, neoepitope load, and transcriptional signatures in the tumor microenvironment (TME) on patients’ overall survival (OS) and progression-free survival (PFS). On this basis, we have identified combinations of tumor characteristics and immune signatures that can strengthen the identification of patients that will likely experience clinical benefit following ICI treatment.

## Material and methods

### Patients

The study cohort contains thirty-two patients with metastatic solid tumors referred to treatment with checkpoint inhibitors, blocking the PD-L1 and PD-1 axis from December 2014 to February 2018. Patients in this cohort were, by the time of the first medical appointment, offered inclusion into the Copenhagen Prospective Personalized Oncology (COPPO) study at the Phase 1 Unit—Rigshospitalet, Copenhagen, Denmark (Tuxen et al., 2014; Tuxen et al., 2018). Prior to treatment initiation, patients was informed about and consented to the possibility of comprehensive genomic analysis, i.e., whole-exome sequencing and RNA sequencing of their tumors. This program is a feasibility study in phase I setting for patients with solid tumors and exhausted treatment options.

Inclusion criteria for immune therapy were defined by the protocols with available slots (EUDRACT number: 2013-002844-10, 2014-002835-32, 2014-002605-38, 2014-000948-14, 2015-003771-30, and 2017-001147-13) and for two patients (pt no. 10 and 20) treatment off-label was given based on high mutational burden (>1,000 non-synonymous mutations). Response to treatment was assessed according to the “response evaluation criteria in solid tumors” (RECIST) 1.1 criteria. For clinical evaluation, we report, the best RECIST response obtained and lasting for at least 2 months.

Fresh Tumor biopsies were primarily taken from metastatic sites, including lymph node and liver. Before the treatment initiation. DNA was purified from blood (germline) and tumor to determine the tumor specific somatic mutations through WES. Three patients were excluded from the analyses, because no pre-treatment biopsy was available (two patients); or no germline WES from blood was available (one patient). Consequently, the presented analyses are based on the remaining 29 patients. Demographic data for these 29 patients can be seen in Table 1. It should be noted that the RNAseq analysis for patient no. 19 did not succeed, but the patient is still included in the data analysis using the microarray data.

**TABLE 1:** Overview of diagnoses, given treatment and response pattern.

Id	Gender	Age	Diagnosis	Treatment	RECIST (best obtained)	Biopsy site	Number of prior treatments
1	Female	64	PAAD	Atezolizumab + Cergutuzumab Amunaleukin	PD	Liver	2
2	Female	62	BRCA	Atezolizumab	PD	Liver	6
3	Female	28	COAD	Atezolizumab + Cergutuzumab Amunaleukin	PD	Lung	3
4	Female	46	READ	Atezolizumab + Cergutuzumab Amunaleukin	PD	Liver	3
5	Female	42	CESC	Atezolizumab + Selicrelumab	PD	Lymph node	6
6	Male	70	PAAD	Atezolizumab + Cergutuzumab Amunaleukin	PD	Lung	2
7	Female	50	CCA-IG	Ipilimumab + Nivolumab	PD	Peritoneum	4
8	Female	51	SKCN	Pembrolizumab	PD	Liver	3
9	Female	43	BRCA	Pembrolizumab	PD	Lymph node	6
10	Male	71	COAD	Nivolumab	PD	Primary tumor	2
11	Male	70	BLCA	Pembrolizumab	PD	Peritoneum	2
12	Female	47	BRCA	Atezolizumab + Selicrelumab	PD	Liver	6
13	Male	61	UC-U	Pembrolizumab	SD	Liver	2
14	Female	38	CESC	Pembrolizumab	PR	Lymph node	4
15	Female	53	CDC-K	Nivolumab	PR	Kidney	1
16	Female	71	BRCA	Atezolizumab	SD	Subcutaneous/Cutaneous	7
17	Female	56	BLCA	Atezolizumab + Selicrelumab	SD	Other	2
18	Female	70	BLCA	Ipilimumab + Nivolumab	PR	Lymphnode	2
19	Male	64	BLCA	Ipilimumab + Nivolumab	CR	Lymphnode	2
20	Male	42	COAD	Pembrolizumab	PD	Subcutaneous/Cutaneous	3
21	Male	74	LIHC	Nivolumab + Relatlimab	CR	Liver	2
22	Male	61	BLCA	Pembrolizumab	PR	Lymph node	1
23	Male	73	READ	Atezolizumab + Cibisatamab	PD	Liver	3
24	Female	63	OV	Atezolizumab + BET inhibitor	PD	Subcutaneous	6
25	Female	41	BRCA	Chemotherapy + Pembrolizumab	PR	Lymph node	3
26	Female	42	BRCA	Chemotherapy + Pembrolizumab	PD	Lymph node	1
27	Female	56	COAD	Atezolizumab + Cibisatamab	PD	Liver	2
28	Female	49	OV	Atezolizumab + BET inhibitor	SD	Lymph node	3
29	Male	67	READ	Atezolizumab + Selicrelumab	PD	Liver	4

The cohort consists of 12 different diagnoses, including; BLCA; bladder urothelial carcinoma, BRCA; breast invasive carcinoma, CCA-IG; Clear cell adenocarcinoma-intern genitalia, CDC-K; Collecting duct carcinoma-kidney, CESC; cervical squamous cell carcinoma and endocervical adenocarcinoma, COAD; colon adenocarcinoma, LIHC; liver hepatocellular carcinoma, OV; ovarian serous cystadenocarcinoma, PAAD; pancreatic adenocarcinoma, READ; rectum adenocarcinoma, SKCN; skin cutaneous melanoma, UC-U; Urothelial carcinoma-Urethra. The given chemotherapy consists of Carboplatin and Gemcitabine. In total, the cohort consists of 10 males and 19 females. The average age of the cohort is 56 years ranging from 28 to 74 years. The average number of treatments prior to the ICI, treatment for this study is 3.2 ranging from 1 to 7 different kinds of treatments.

### Molecular analysis of tissue biopsies

Biopsies stored in RNAlater (Sigma-Aldrich) were used for comprehensive molecular profiling. Briefly, DNA and RNA were isolated using AllPrep DNA/RNA kit (Qiagen). Blood samples were collected in EDTA tubes, and genomic DNA was extracted using a Tecan automation workstation (Promega). Molecular profiling consists of whole-exome sequencing (Illumina platform) and mRNA expression arrays (Human U133 Plus2.0, Affymetrix).

DNA libraries were prepared from 200 ng of DNA. Fragmentation was done on Covaris S2 (Agilent) to approximately 300-bp fragments, and adaptor ligation was done using KAPA HTP Library Preparation Kit. Exomes were enriched with SureSelectXT Clinical Research Exome kit (Agilent). Sequencing was carried out as paired-end sequencing, aiming at an average coverage of 50–100x using the HiSeq2500 and NextSeq500 platforms from Illumina. RNAseq libraries were prepared from 100 ng of total RNA using the Total RNA-Seq library Prep Kit (Illumina). Sequencing was done on the HiSeq2500 and NextSeq500 platforms.

Purified RNA was immediately analyzed on microarrays. RNA was reverse transcribed and used for cRNA synthesis, labeling, and hybridization with GeneChip^®^ *Human Genome U133 Plus 2.0* Array (Affymetrix) according to the manufacturer’s protocol. The arrays were washed and stained with phycoerythrin conjugated streptavidin using the Affymetrix Fluidics Station 450, and the arrays were scanned in the Affymetrix GeneArray 3,000 7G scanner to generate fluorescent images.

### Next-generation sequencing data analysis

WES and RNAseq data were processed according to the Genome analysis tool kit (GATK) best practice guidelines for somatic variant calling (Van der Auwera et al., 2013). Raw reads from both were quality trimmed using the wrapper tool Trim Galore 0.4.0 (Krueger, 2021), combining Cutadapt (Martin, 2011) and FastQC (Andrews, 2010) trimming reads to an average phred score of 20 and a minimum length of 50 bp. Reads were aligned to the human genome (GRCh38) using the Burrows-Wheeler Aligner (Li and Durbin, 2009) version 0.7.16a with default mem options and with a read-group provided for each sample, thereby ensuring compatibility with the following steps. Reads were sorted using Samtools 1.6 (Li et al., 2009). Duplicated reads were marked using the Picard-tool version 2.9.1 MarkDuplicates. To reduce false positive variant calls, base recalibration was performed with GATK version 4.0.1.1. SNV and indel calls were made using GATKs build-inn version of MuTect2 (Cibulskis et al., 2013) designed to call somatic variants for both single nucleotide variants (SNVs) and indels from matched tumor and normal samples. HLA alleles of each patient were inferred from the WES data using OptiType 1.2 (Szolek et al., 2014) with default settings after filtering the reads aligning to the HLA region with RazerS version 3.4.0 (Weese et al., 2012) Kallisto 0.42.1 (Bray et al., 2016) was used to determine the gene expression from RNAseq data.

### Differential expression analysis and gene set enrichment analysis

Raw microarray data were imported into R and normalized by the Robust Multi-array Average (RMA) algorithm. The “hgu133pLus2. db” package version 4.1.0 was used to translate between probe set IDs and Human Gene Organization (HUGO) gene names. The “limma” package (Smyth, 2005) (version 3.5.3) was used to test for differential expression between groups with 770 selected genes from a pan cancer gene panel (Cesano, 2015). *p* values were adjusted using the method of Benjamini and Hochberg (BH). The package “ComplexHeatmap” version 2.13.1 was used to create heat maps (Yu et al., 2012; Gu et al., 2016) of the differential expressed genes with adjusted *p*-value <0.05 and log foldchange >0.5 and log foldchange < −0.5. Gene Set Enrichment Analysis (GSEA) is made from the differential expression analysis results in R with cluserProfiler (Yu et al., 2012) version 4.0.5 and enrichplot (Yu, 2021) version 1.13.2 with Gene Ontology pathway database. CYT was calculated as the geometric mean of the gene expression of granzyme A (GZMA) and perforin (PRF1) both for microarray and RNAseq.

### Assessment of TMB and neoepitope load

The total tumor mutational burden of all mutations acquired in each tumor was assessed by counting each entry passing the filtering criteria of GATK4’s MuTect2 output VCF file. This VCF file was given as input to the neoepitope predictor, mutant peptide extractor and informer (MuPeXI) 1.2.0 (Bjerregaard et al., 2017) together with RNAseq expression values obtained from Kallisto in transcripts per million (TPM) and the HLA alleles detected by OptiType. The output neopeptides were selected based on the expression level of the gene of origin (>0.1 TPM) and the predicted MHC binding eluted ligand percentile rank (EL %Rank) score <2, evaluated by NetMCHpan 4.0 (Jurtz et al., 2017). The number of selected potential neoepitopes was used as the neoepitope load. Additionally, TMB of non-synonymous mutations were determined from the MuPeXI output logfile summarizing peptides originating from missense variant mutations, in-frame insertions, and deletions, together with frameshift mutations. Mutation types were determined by Ensembl’s variant effect predictor (VEP) version 87 (McLaren et al., 2016) as a dependency of MuPeXI.

### Determination of T cell diversity by CDR3 sequence identification from RNAseq

MiXCR (Bolotin et al., 2015) version 2.1.1 was used to determine complementarity-determining region 3 (CDR3) sequences from bulk RNAseq data with the optimized setting for this specific purpose (Brown et al., 2016). The quality trimmed reads from RNAseq were used as input following MiXCR’s identification of specific clone identification from the IMGT database (Smyth, 2005) reference of known CDR3 sequences, together with the clone count of each clone detected referring the reads aligning to this specific clone of the CDR3 reference library. Shannon entropy (Shannon, 1948) was calculated as a T cell diversity measurement based on the number of unique CDR3 sequences, or T cell clones, detected in the individual patient (Stewart et al., 1997).

### Survival analysis

Patients are separated into two groups compared to the median of the observed value. For a patient to be included in the “high” category in the combination of biomarkers, that patient must have values above or equal to the median for all biomarkers within the combination, while the remaining patients are then placed in the “low” category. Additionally, we included combinations of whether patients were “high” in three or more and two or more of any of the four investigated biomarkers compared to the remaining patients. Hazard ratios with the corresponding confidence intervals were calculated for each biomarker and all combinations, whereas the single and the best combinations were using the suvminer packages version 0.4.9 (Alboukadel Kassambara et al., 2021) and survival packages version 3.5.0 (Therneau and Grambsch, 2000) in R studio version 4.1.1.

### Statistical analysis

Since the data analyzed in this study is not normally distributed, an unpaired Mann-Whitney/Wilcoxon rank-sum test was used, with normal approximation using continuity correction of the calculated *p*-value to calculate and determine statistically significant differences between groups using R statistical software version 4.1.1.

### Validation cohort

The validation cohort consists of 24 metastatic urothelial carcinoma (mUC) patients (Holm et al., 2022). WES and RNAseq are preprocessed in the same manner as the sequencing data from this study, and a detailed description can be found in (Holm et al., 2022). Shortly, variants are called with GATK 3.8, and TPM from each gene was found by Kallisto alignment.

## Results

### Patient cohort and clinical outcome

We investigated different biomarkers for their potential to identify patients with a favorable clinical outcome in a diverse cohort of 29 cancer patients with 12 different tumor indications treated with 10 different ICI combinations. All treatments included blocking the PD-1 to PD-L1/PD-L2 interaction (Table 1). In total, 15 patients were treated with PD-1 ICI (Pembrolizumab, or Nivolumab) and 14 patients with PD-L1 ICI (Atezolizumab), often in combination with other treatment modalities (e.g., Ipilimumab, chemotherapy or antibodies blocking other immune checkpoint molecules).

**FIGURE 1:**
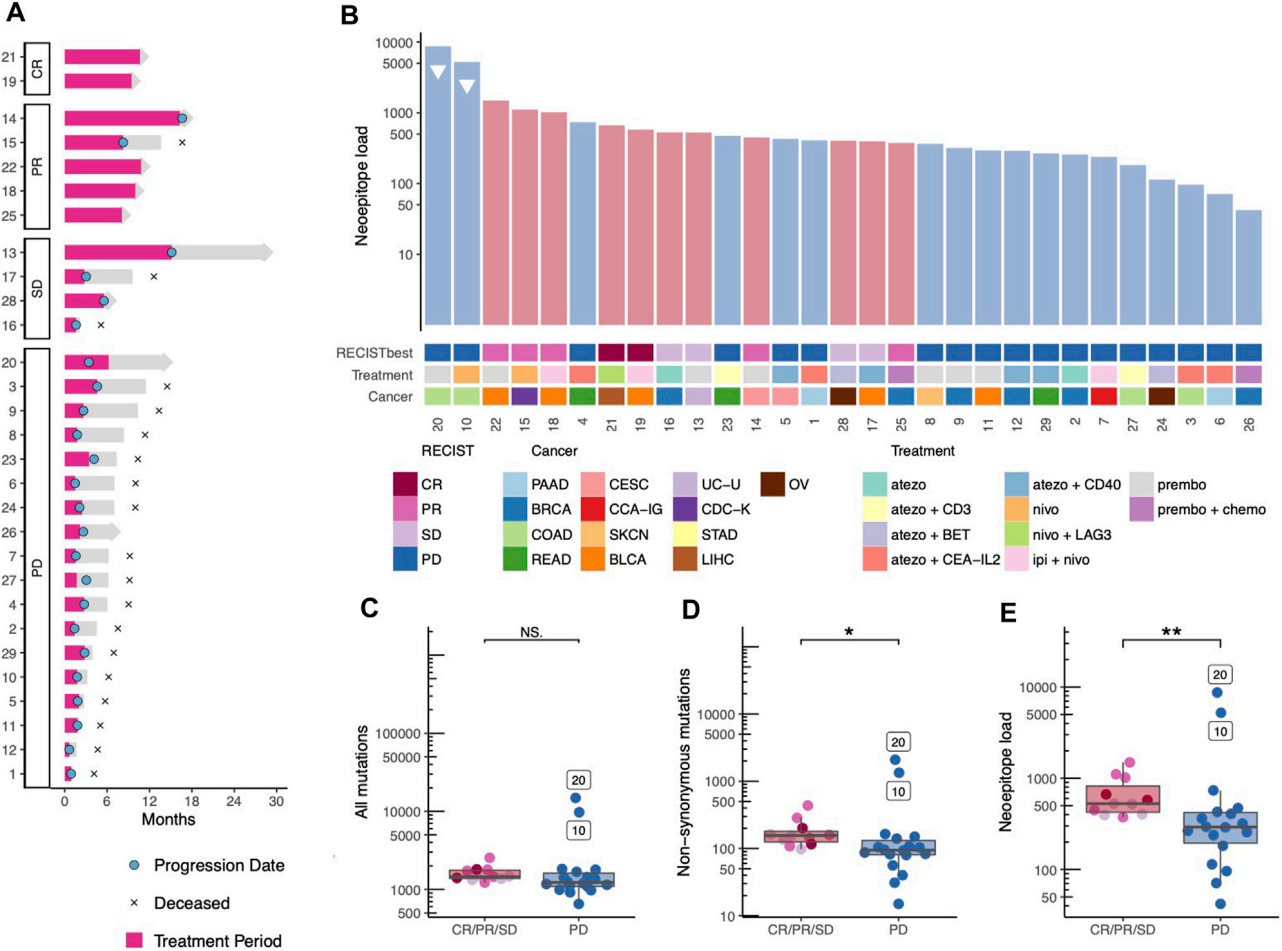
Treatment trajectory, Tumor mutational bourdon and neoepitope load. **(A)** The treatment trajectory for each patient is plotted according to response, with annotations of key dates, treatment period is highlighted in pink. **(B)** Each patient is represented in a barplot of predicted neoepitope load with annotations according to best obtained RECIST criteria, cancer type, and immunotherapy treatment combination. Patients with high microsatellite instability are annotated with a triangle. BLCA; Bladder Urothelial Carcinoma, BRCA; Breast invasive carcinoma, CCA-IG; clear cell adenocarcinoma - intern genitalia, CDC-K; collecting duct carcinoma-kidney, CESC; Cervical squamous cell carcinoma and endocervical adenocarcinoma, COAD; Colon adenocarcinoma, LIHC; Liver hepatocellular carcinoma, OV; Ovarian serous cystadenocarcinoma, PAAD; Pancreatic adenocarcinoma, READ; Rectum adenocarcinoma, SKCN; Skin Cutaneous Melanoma, STAD; Stomach adenocarcinoma, UC-U; Urothelial carcinoma-Urethra. **(C-E)** The mutation and neoepitope load are colored according to best obtained RECIST criteria and grouped by progressive vs. non-progressive disease statistic test are med with Wilcox test. **(C)** Total mutation burden (*p*-value = 0.069, Wilcoxon rank sum test). **(D)** Non-synonymous mutations (*p*-value = 0.012, Wilcoxon rank sum test) and **(E)** number of predicted neopeptides—referred to as neoepitope load (*p*-value = 0.009, Wilcoxon rank sum test).

Response to therapy was evaluated using RECIST 1.1 as the best obtained response was reported. The patient cohort included two complete responders (CR), five patients with partial response (PR), four with stable disease (SD), and 18 with progressive disease (PD). In this study, we investigate the differences between patients with PD and non-progressive disease (CR, PR, SD). The cohort included twenty deceased patients due to progressive disease, while ten patients were still under observation by the end of the analysis (five of these with no signs of disease progression) (Figure 1A). All patients had previously been treated with at least one and up to seven prior treatment lines, none of which were immunotherapies (Table 1). It is noteworthy that two patients obtained CR, and both received a combination of checkpoint inhibition treatments, nivolumab and ipilimumab, and nivolumab and relatlimab (LAG-3), respectively. When looking into the different checkpoint inhibition combinations used and the cancer type of the patients included, no obvious difference in clinical response related to treatment regimen nor cancer type was observed (Figure 1B). When comparing the survival of the patients receiving monotherapy to those receiving combination therapy, no difference was observed (Supplementary Figure S1). It should be noted, however, that this study is not intended to identify the difference between tumor or treatment indications since each tumor type and treatment strategy are scarcely represented.

### Neoepitope load is associated with non-progressive disease

First, we investigated biomarkers known to influence overall survival in uniform cohorts of one cancer type including the TMB and the neoepitope load. When ranking the 29 patients according to the predicted neoepitope load, we observed that patients do cluster based on clinical outcome, progressive compared *versus* non-progressive disease (Figure 1B). Interestingly, the two patients with colon cancer with microsatellite instability (MSI) were found in the PD patient group (patient no. 10 and 20), despite their very high neoepitope load. When comparing the two patient groups, progressive disease vs. non-progressive disease, no significant difference between was observed in the TMB for all mutations identified (Figure 1C), but we did observe a significant difference (*p*-value = 0.069) in TMB for non-synonymous mutations only (Figure 1D), and the prediction of their neoepitope load provided even further separation (*p*-value = 0.009) between the two patient groups (Figure 1E).

### Selected T cell signatures identifies patients with treatment benefit

**FIGURE 2:**
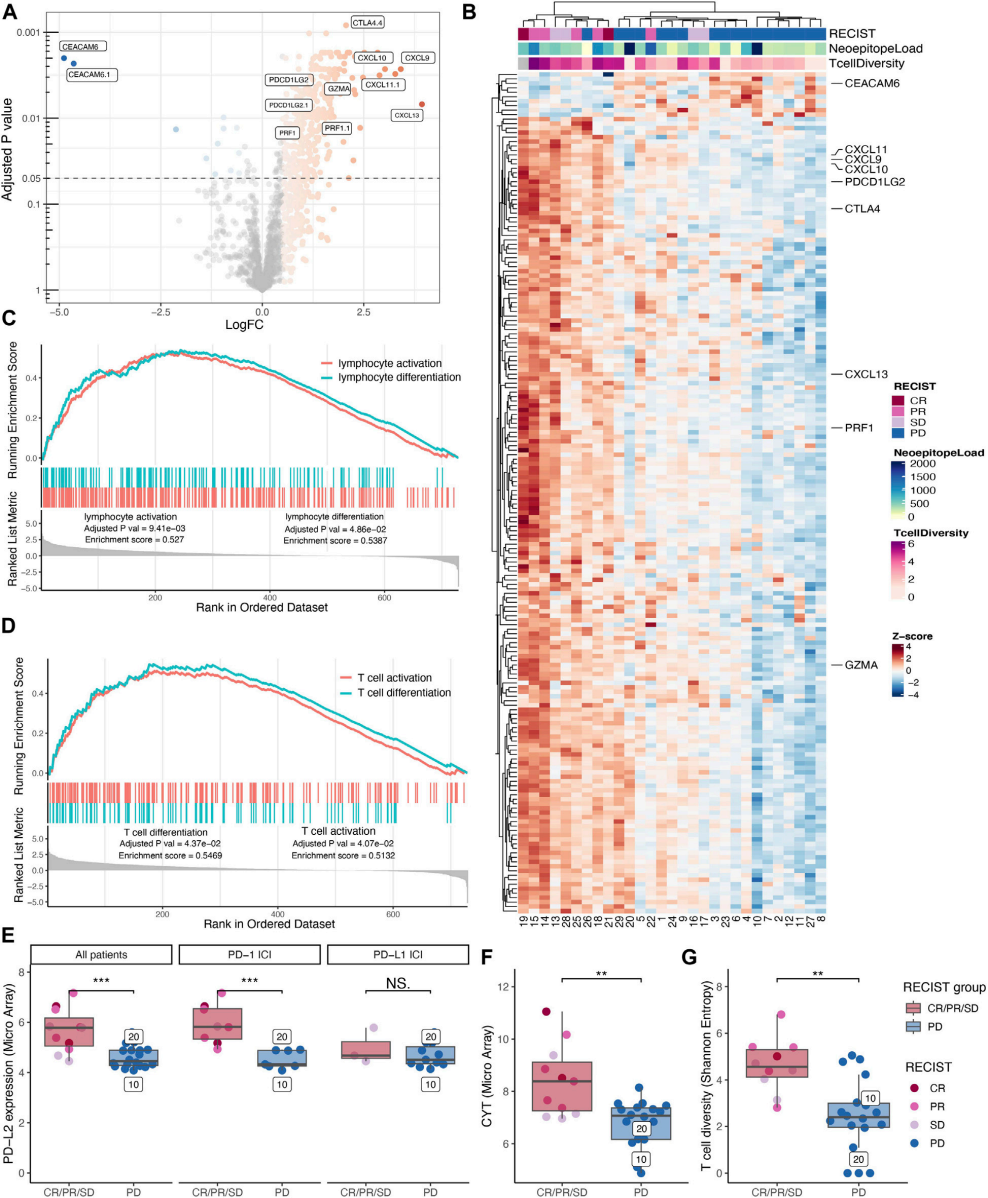
Immunological transcriptional profiling. Differential gene expression analysis between patients with progressive disease vs. non-progressive disease displayed as a volcano plot. **(A)** Shoving all probes extracted from the 770 gene PanCancer Immune Profiling Panel colored according to log foldchange (LogFC) for probes with an adjusted *p*-value below 0.05 and LogFC above 0.5 and below minus 0.5. Probes with the highest variety in LogFC and lowest *p*-values are highlighted together with probes for PRF1, GZMA, and PDCDLG2. **(B)** The mean expression of the significant probes was gathered to reveal 188 significantly differentially expressed genes with z-scores displayed in a double clustered heatmap annotated with best-obtained RECIST criteria, neoepitope load, and T cell diversity (Shannon Entropy). **(C+D)** Significantly gene ontology (GO) pathways enriched in the non-progressive group from the gene set enrichment analysis. **(C)** Lymphocyte activation and lymphocyte differentiation. **(D)** T cell activation and T cell differentiation. **(E-H)** Comparing non-progressive with progressive disease patients and the statistic test are made with Wilcoxon rank sum test. **(E)** Expression of PD-L2 was found to be significantly higher in patients with the non-progressive disease (*p*-value = 0.001). However, when separating PD-L2 expression into patients receiving PD-1 immune checkpoint inhibition (ICI) and those who have received PD-L1 ICI only a significant difference in PD-L2 expression can be found for the PD-1 ICI treated patients (*p*-value = ${3.1∙10}^{-4}$ ) and not for those who have been treated with PD-L1 ICI (*p*-value = 0.456). **(F)** The same was found for the cytolytic value (CYT), measured as the geometric mean of granzyme A and perforin (*p*-value = 0.003). **(G)** T cell infiltration analysis identifying CDR3 sequences from bulk tumor RNAseq data showed that the patients benefiting from treatment had a significantly higher T cell diversity measured by the Shannon Entropy (*p*-value = 0.002).

To investigate whether distinct gene signatures differentiate the patients with progressive disease vs. non-progressive disease, we performed a differential expression analysis of a 770-pan-cancer-immune-related-gene-panel from both the expression array data and the RNAseq data. Due to higher sensitivity, we display the analysis of the microarray data in the main figures. The differential expression analysis revealed that 322 microarray probes, (Figure 2A), were differentially enriched between the two groups. Following double cluster analysis of the mean of these enriched probes, they condense into 188 genes. This gene signature tends to cluster according to disease outcome (RECIST), with a particular clustering of the patients with non-progressive disease vs. progressive disease (Figure 2B). We note that the two MSI colorectal cancer patients with the highest neoepitope load are clustered together with the other progressive disease patients according to the gene enrichment signature from the TME, indicating that an unfavorable tumor microenvironment may override the role of the high TMB in promoting tumor foreignness and immune recognition (Figure 2B). To identify biological pathways of interest, a gene set enrichment analysis (GSEA) was performed, revealing lymphocyte differentiation (Figure 2C) and more specifically, T cell differentiation pathways (Figure 2D) to be significantly enriched in the group of patients with non-progressive disease.

To investigate potential biomarkers from the tumor microenvironment (TME) that could be used to identify patients who will benefit from treatment, we investigated the intratumoral T cell presence and associated factors immunological signatures. Based on two different probe sets we find PD-L2 (gene synonym PDCD1L2), highlighted in Figure 2A, to be preferentially expressed in the TME from patients with non-progressive disease. When evaluating the expression level of PD-L2 in the individual patients, we observed a significantly higher expression (*p*-value = 0.001) in patients with non-progressive disease compared to progressive disease patients (Figure 2E, left) and confirmed in RNAseq (Supplementary Figure S2A). The ICI treatment with anti-PD-Ll blocks the interaction between PD-1 and PD-L1 but have no direct effect on the binding of PD-L2 to PD-1. Hence, we re-evaluated the PD-L2 expression data by splitting patients into two groups depending on their treatment modality including anti-PD-1 or anti-PD-L1, respectively. As a consequence of this split, the number of subjects is low, and the PD-L1 treated cohort includes only SD patients in the “non-progressor” group. Albeit the data suggest that the predictive effect of PD-L2 expression is stronger in the anti-PD-1 treated patients (Figure 2E, middle + right).

We investigated Cytolytic activity (CYT) as a biomarker in this cohort, which previously has been described as a biomarker for response to immunotherapy (Rooney et al., 2015), and found that CYT has a significantly higher expression (*p*-value = 0.003) in patients with non-progressive disease (Figure 2F), further confirmed in the RNAseq (Supplementary Figure S2B). Further, we examined the T cell infiltrate by the T cell diversity, and we found that the patients with non-progressive disease have a significantly higher (*p*-value = 0.001) T cell diversity compared to patients with progressive disease (Figure 2G). The T cell diversity correlates with CYT (person correlation = 0.744) as both strategies quantify the T cell infiltrate (Supplementary Figure S2C). We found that both neoepitope load, PD-L2 expression, CYT, and T cell diversity can be used as potential biomarkers to distinguish non-progressive disease patients from progressive disease patients. By combining predicted neoepitopes with PD-L2 expression, non-progressive disease patients are clustered in the high-high quadrant split by the median of each value (Supplementary Figure S2D). The same pattern can be observed with CYT and PD-L2, as well as neoepitope load and CYT (Supplementary Figures S2E, F). Consequently, these may be interesting features for a combined biomarker.

### Combined biomarkers improve survival probability

**FIGURE 3:**
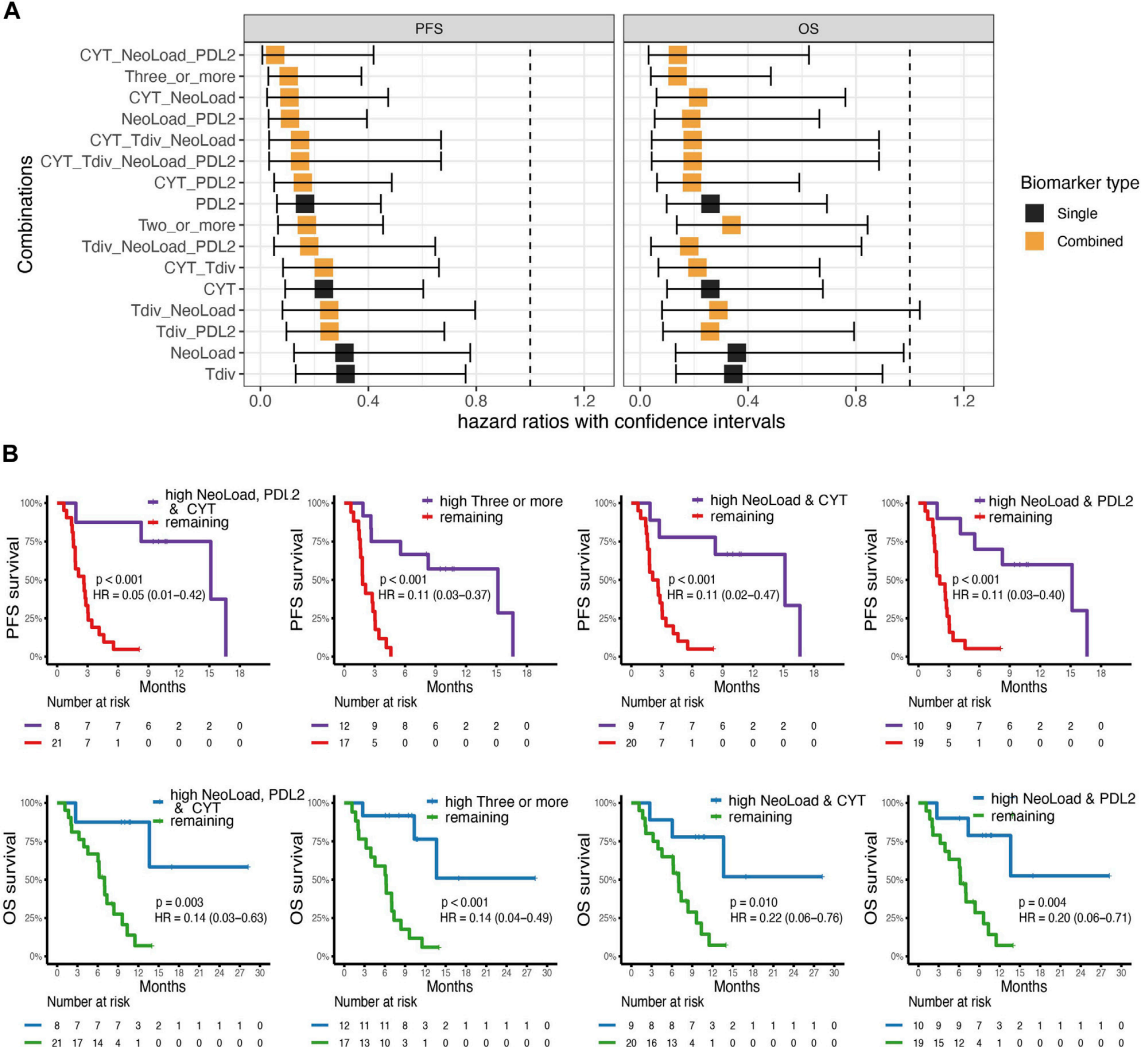
Survival analysis. **(A)** Cox-regression analysis for four suggested biomarkers PD-L2 expression (PDL2), Cytolytic Activity (CYT), T cell diversity measured by the Shannon Entropy (Tdiv), and Neoepitope load (NeoLoad), and all combinations of these four biomarkers where “high” were defined by values above or equal to the median. On the *x*-axis, the square indicates the hazard ratio (HR) and the error bars indicate the confidence interval. The color indicates whether the biomarker is a single biomarker (in black) or a combined biomarker (in orange). Left; the analysis for Overall Survival (OS) and right; the analysis for progression-free survival (PFS). **(B)** Kaplan-Meier curves for the top four combinations obtained from the analysis made from the cox-regression analysis with survdiff log-rank test for *p*-values. Left; a combination of high NeoLoad, high PD-L2 expression, and high CYT showed significantly improved survival probability for both PFS (*p* = ${2∙10}^{-4}$ ) and OS (*p* = 0.003). Middel-left; patients with high in three or more signatures, also had increased survival probability both for PFS (*p* = ${4.4∙10}^{-5}$ ) and OS (*p* = ${3.7∙10}^{-4}$ ), middel-right; patinets with high NeoLoad and CYT, respectively PFS (*p* = ${4.6∙10}^{-4}$ ) and OS (*p* = 0.010). Right; patients with high NeoLoad, and PDL2, respectively, also had increased survival probability both for PFS (*p* = ${1.0∙10}^{-4}$ ) and OS (*p* = 0.004).

To examine the probability of four different suggested biomarkers, Neoepitope load (NeoLoad), PD-L2 expression (PDL2), CYT, and T cell diversity (Tdiv), and combinations hereof to identify patients with a favorable clinical outcome, we applied cox regression to analyze the association with OS and PFS. We analyzed all biomarkers individually and all possible combinations of the biomarkers. For the analysis, two groups (“high” and “low”) were established for each biomarker split by their median value. From this analysis, we found that the best combination of biomarkers according to the hazard ratio for PFS and OS was obtained when CYT, NeoLoad, and PDL2 were combined (Figure 3A) with hazard score of 0.05 (0.01–0.42) and 0.14 (0.03–0.63), respectively. The four best biomarker combinations were illustrated using Kaplan-Meier curves (Figure 3B). All four combinations can significantly separate favorable from unfavorable patient outcome, based on PFS and OS, respectively. All individual biomarkers were illustrated with Kaplan-Meier curves (Supplementary Figure S3).

### Validation cohort

**FIGURE 4:**
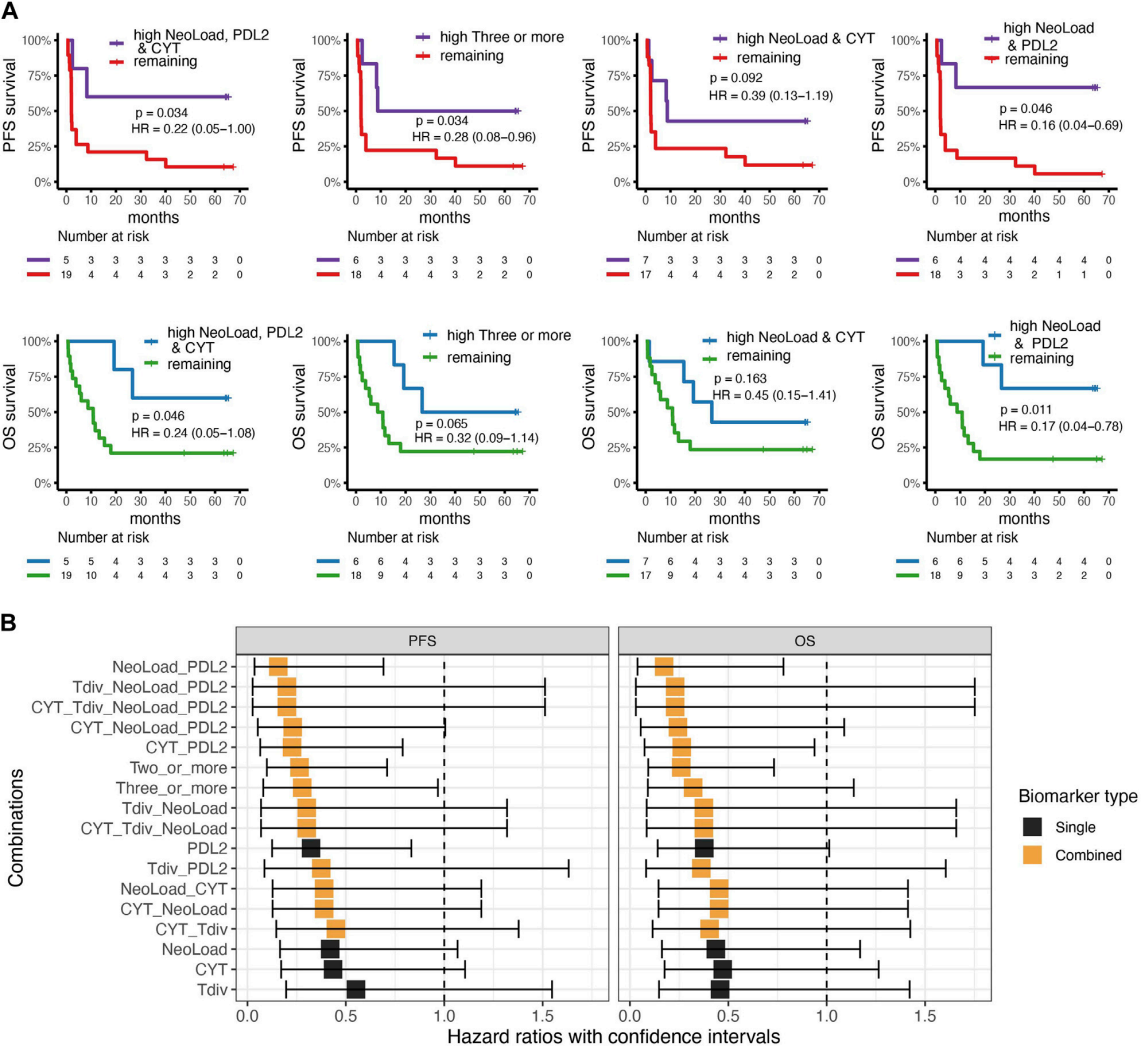
Validation cohort. Cox-regression and survival analysis from the validation cohort with survdiff log-rank test for *p*-values. **(A)** Kaplan-Meier curves for best four combinations found in the cohort from the basket trail with progression-free survival (PFS) in the top and overall survival at the bottom where high indicates values above or equal to the median for the cohort. Left; comparing patients with high neoepitope load (NeoLoad), high cytolytic activity (CYT), and high PD-L2 expression (PDL2) to the remaining patients which showed that patients with high NeoLoad, CYT, and PDL2 had a higher PFS and OS probability (*p* = 0.034 and 0.046). Middle-left; three or more signatures as high compared to the remaining where PFS is significant in PFS (*p* = 0.034) and non-significant in OS (*p* = 0.065). Middel-right; patients with high NeoLoad and CYT, respectively PFS (*p* = 0.092) OS (*p* = 0.163). Right; patients with high NeoLoad and PDL2, respectively, compared to the remaining patients resulted in a significantly higher PFS probability for the patients in the “high” group (*p* = 0.046) and a significantly higher OS probability (*p* = 0.011). **(B)** Cox-regression analysis of the four different suggested biomarkers including PD-L2 expression (PDL2), Cytolytic Activity (CYT), T cell diversity measured by the Shannon Entropy (Tdiv), and Neoepitope load (NeoLoad) and all their combinations illustrated for overall survival (OS) and Progression-free survival (PFS). Black squares indicate the hazard ratio, whereas the error bars indicate the confidence interval. The color indicates whether the biomarker is a single biomarker (in black) or a combined biomarker (in orange).

WES and RNAseq from a cohort of 24 metastatic urothelial carcinoma (mUC) patients all treated with anti-PD-L1 ICI (Holm et al., 2022), were used to validate the investigated biomarkers. The first combination with NeoLoad, CYT, and PDL2 was significant in identifying patients with longer PFS (*p*-value = 0.034) and OS (*p*-value = 0.046) (Figure 4A, left). The combination of any three or more biomarkers categorized as high also showed a significant difference in PFS (*p* = 0.034) and borderline non-significant separation in OS (*p* = 0.65) (Figure 4A, middle-left). Thus, while confirming the findings from the basket trial, the CYT value seemed to play less of a role in the mUC cohort (Figure 4A, middle-right), and where the combination of only NeoLoad and PDL2 provided equally good separation related to both PFS (*p*-value = 0.046) and OS (*p*-value = 0.011) (Figure 4A, right). Neoepitope load, CYT, T cell diversity, and PD-L2 were also individually investigated for their predictive value in the validation cohort, but no significance was observed based on the single parameters (Supplementary Figure S4). Cox-regression analyses were conducted for the validation cohort, as in the primary cohort, using all suggested biomarkers and all combinations (Figure 4B). Again, this demonstrated that NeoLoad and PDL2 was the best combination to predict patient’s outcome, related to both PFS and OS.

Overall, both the primary cohort and the validation cohort agree that a combination of biomarkers was better at predicting survival than a single biomarker alone. Summarizing based on the two cohorts, patients with high neoepitope load, and high PD-L2 expression, potentially combined with high CYT, resulted in a significantly improved survival probability.

## Discussion

Despite the recent success of immunotherapy, the objective response rate rarely reaches >50% (Yarchoan et al., 2017; Chowell et al., 2022). Hence, there is a need to segregate patients likely to respond to treatment and understand the biological basis of treatment success and failure.

In this study, we explore the impact of neoepitope load, PD-L2 expression, cytolytic transcriptional signature (CYT), and T cell diversity (Tdiv) as biomarkers for predicting the outcome of ICI treatment. Our data suggest that individual parameters can serve as biomarkers to distinguish progressive from non-progressive disease patients, but are rarely sufficient to predict treatment benefit across broader patient cohorts, where tumor heterogeneity may be substantial (Dagogo-Jack and Shaw, 2018; Liu et al., 2018). Here, we observe that a single parameter, the neoepitope load (NeoLoad) obtained a hazard score of 0.31 and 0.36 respectively, for PFS and OS, but the two MSI patients with the highest NeoLoad represents outliers, that did not benefit from the treatment, despite their high NeoLoad. Using CYT alone, a hazard score of 0.24 and 0.26, respectively, for PFS and OS was achieved, and in this case MSI patients belonged to the “low” category, in accordance to their clinical outcome. Similar observation can be obtained by observing T cell diversity alone. These case stories demonstrates that single biomarkers rarely covers the immune-response relevant characteristics of a broader cohort. Consequently, the three single biomarkers (NeoLoad, CYT, and Tdiv) revealed among the lowest predictive value when used independently, both in main cohort and the validation cohort (Figure 3A; Figure 4B). PD-L2 as a single biomarker obtained the lowest hazard score compared to the other single biomarkers, but the predictive value was further improved by pairing with other characteristics, NeoLoad, CYT or Tdiv. When using PDL2 as a single biomarker some of the PD patients were placed in the “high” group and hence be wrongly predicted (Supplementary Figure S2). All these observations suggests that a combination of biomarkers is better suited to cover the patient- and tumor diversity, and consequently provide a more robust predictive value for patient selection. As such, the combination of NeoLoad and PD-L2, and possible CYT expression could significantly identify patients with clinical benefit in both our primary and validation cohorts. Additionally, the selected biomarkers provided a significant separation of patients, measured by their progression free survival and overall survival, which may relate to both a predictive and prognostic value of such immune signatures.

The clinical study examined here was conducted as a so-called “basket” trial, where patients with numerous different cancer types are subjected to the same clinical strategy and our data suggest that the identified biomarkers can be used for patient stratification across different tumor types and ICI treatments. This clinical practice relates to the increased understanding that the characteristics associated with clinical responses to immunotherapy are often tumor-type agnostic and are defined by the immune and inflammatory signature and foreignness of both the tumor cells and the TME. Such characteristics can vary in different tumor types, leading to different response rates to ICI, but the mechanism on interaction and influence on treatment is most often tumor agnostic (Taube et al., 2014; Chowell et al., 2022).

Our data demonstrate that combining biomarkers is more robust than using a single biomarker, and analysis from the validation cohort supports those findings. As an illustrative case, patient no. 26, initially categorized as a partial responder progressed quickly after the first treatment (therefore not meeting the requirement of sustained a RECIST response for at least 2 months after treatment initiation). This patient turned out to have a gene expression profile corresponding to non-progressors (Figure 2B) but displayed the lowest detected neoepitope load of this cohort. This example suggests that not only is the right immunologic gene expression profile of the tumor tissue important but also a sufficient neoepitope load is needed, most likely for the tumor cells to be “visible” to the immune system. On the other hand, a high neoepitope load alone is not sufficient, as seen for the PD MSI patients no. 10 and 20, who, despite having the highest neoepitope load of the cohort (Figure 1D), displayed an immunological gene signature comparable to the progressive disease patients (Figure 2B). Interestingly, previous data has demonstrated high response rates to checkpoint inhibition therapy in patients with MSI tumors (Le et al., 2015), and based on such data checkpoint inhibition therapy is approved for the treatment of all MSI cancers, despite origin (Lemery et al., 2017). The two MSI-high cases included in our cohort did not respond to therapy, and illustrates that additional biomarkers are needed to identify those patients where checkpoint inhibition is not sufficient even in this category of patients with a high TMB.

Taken together, our study demonstrates the need to combine different markers rather than relying on isolated markers when selecting patients likely to benefit from ICI. The interactions determining how T cells recognize and, ultimately, kill cancer cells are the result of myriad processes, and modulating immune response by check-point inhibition is only a single trigger in a larger biological cascade. Attempts to establish a multiparametric system comprising the mechanisms behind these interactions, such as the cancer immunogram (Blank et al., 2016) have been made, but the applicability in a clinical setting requires algorithms capable of managing not only large-scale data but also different types of data, and how to balance the input of the different parameters. The idea of combining neoantigen and immune signatures as a biomarker has been suggested in melanoma patients treated with adoptive cell transfer (ACT) (Lauss et al., 2017). Machine learning strategies with multiple biomarkers have also been used to predict patient outcome for treatment with ICI but these machine learning algorithms needs large-scale data to make valuable predictions (Acharjee et al., 2020) and are therefore not used in this study, where the patient cohort is relatively small. The strategy applied here has the limitation that by separating patients into two groups with respect to high and low expression of certain gene signatures, some patients display borderline characteristics, and hence may be false categorized. The strength of machine learning approaches is their capacity to address a continuum of expression and a large number of parameters, thereby avoiding the need for strict and pre-defined cut-off values. But as mentioned, this requires very large datasets to avoid overfitting results and to capture the variability that is observed within and across cancer patient cohorts. Access to biological and clinical data from such large cohorts, where sequencing data from different biological specimens are available at high quality is still a major limitation towards developing such algorithms.

PD-L2 interaction with PD-1 inhibits T cell activation (Latchman et al., 2001). Furthermore, the function and importance of PD-L2 have recently been investigated and suggested as an important target for cancer (Solinas et al., 2020). We see a correlation between high expression of PD-L2 and better survival probability both for patients treated with anti-PD-1 and anti-PD-L1. This trend could be explained by high expression of PD-L2 in TME, being a signature of immune activity in the tumor site. This agrees with a previous study that showed a positive correlation between high PD-L2 expression and lymphocytic infiltration and improved overall survival (Obeid et al., 2016). Furthermore, for patients treated with anti-PD-1, the PD-L2 molecule is directly involved in the immunosuppressive axis that is being blocked by treatment.

The biomarkers suggested in this study, is identified from the analysis of sequencing data and microarray data. Although two datasets have been used, further validation of such signatures using targeted strategies, such as qPCR, are warranted for validation. Additionally, single-cell experiments would be of interest, especially considering the PD-L2 expression, to determine whether the expression arises from tumor cells or other cells in the TME.

A challenge associated with most of the biomarkers currently identified as relevant for the prediction of response to immunotherapy, including those described here, is that they require the availability of tumor material. Future initiatives are heading towards an understating of how susceptibility to immunotherapy can be evaluated by studying circulating tumor cells (CTCs) and circulating tumor DNA (ctDNA) in peripheral blood. Such material might be useful to determine the neoepitope load if sufficiently representative of the tumor.

In conclusion, this study adds to the potential impact of PD-L2, neoepitope load, CYT, and T cell diversity as potential biomarkers. Data from our study and the validation cohort suggest that PD-L2 and neoepitope load both with and without CYT significantly predict patient survival. Due to the small sample size, our results need further validation in larger cohorts.

## Data Availability

The Capital Region of Denmark do not allow sequencing data being reposited in open repositories, however all data will be available upon request to the authors, based on an individual data sharing agreement. Related to our validation cohort: All WES and RNAseq data is available upon application at dbGaP at https://www.ncbi.nlm.nih.gov/projects/gap/cgi-bin/study.cgi?study_id=phs001743.v1.p1. GRCh38 reference genome is available at https://www.ncbi.nlm.nih.gov/assembly/GCF_000001405.39. All other relevant data are available from the authors upon request. A description of covariate data is previously published (Snyder et al., 2017).
