# Supplementary material for: Neoepitope load, T cell signatures and PD-L2 as combined biomarker strategy for response to checkpoint inhibition immunotherapy
Source: Front Genet. 2023 Mar 23;14:1058605. doi: 10.3389/fgene.2023.1058605 (PMC10076713; doi:10.3389/fgene.2023.1058605)
Supplement: Supplementary file 1 [file Presentation1.pdf]

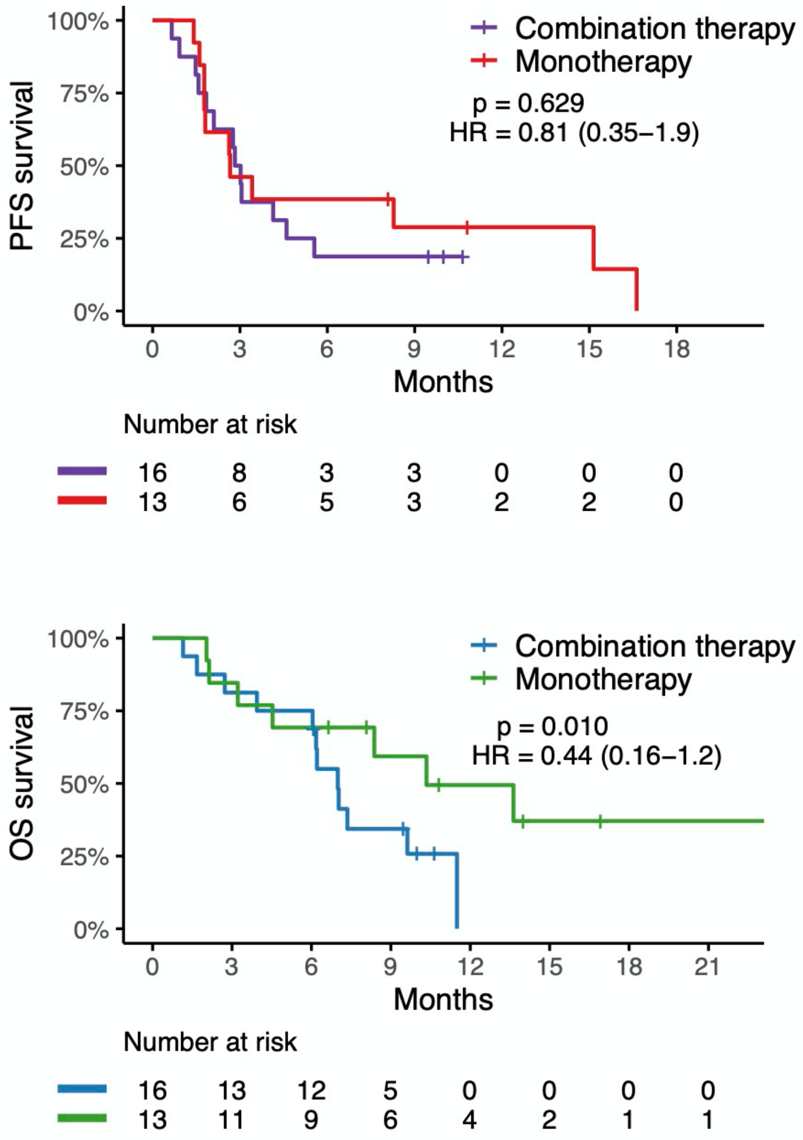

Supplementary Figure 1. Survival curves comparing monotherapy and combination therapy. All patients received treatment with immune checkpoint inhibitors (ICI), and some of the patients received ICI in combination with other therapies. When comparing the survival time of the patients receiving monotherapy with those receiving combination therapy, no difference in either PFS or OS can be observed. Hazard ratio and p-values are calculated using cox-regression with survdiff log-rank test for p-values.

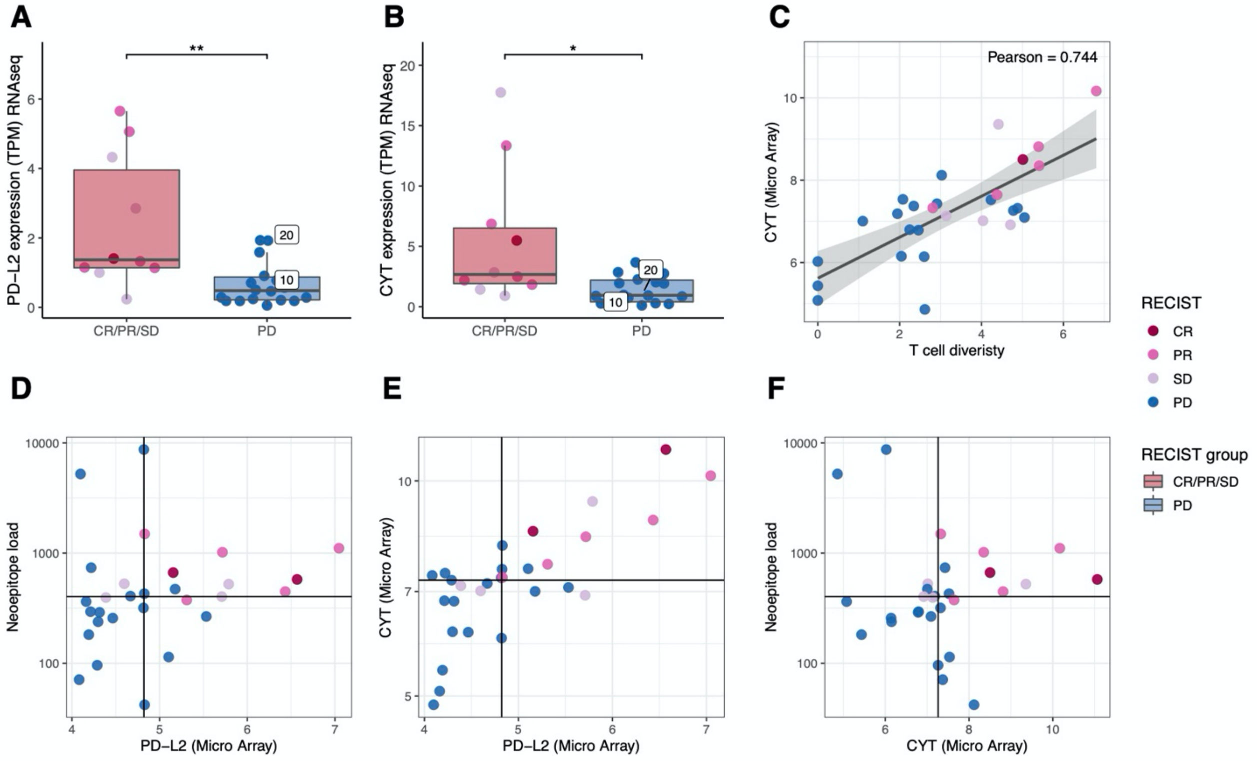

Supplementary Figure 2. Biomarker from RNAseq, T cell signature correlation, and biomarker combinations. **A-C)** The expression from CYT and PD-L2 were validated in RNAseq where the non-progressive group (CR/PR/SD) was compared to the progressive group (PD) group. **A)** PD-L2 expression from RNAseq (TPM) (p-value = 0.003). **B)** Cytolytic activity (CYT) calculated from RNAseq data (p-value = 0.014). **C)** CYT from RNAseq and T cell diversity with correlation (Pearson correlation = 0.744), colored by RECIST. **D)** Neoepitope load vs PD-L2 expression showed that non-progressive disease patients were gathered in the upper right corner with above median expression in both. **E)** same observation by observing PD-L2 expression combined with CYT. **F)** Neoepitope load vs. CYT also showed clustering of non-progressive patients in the upper right corner.

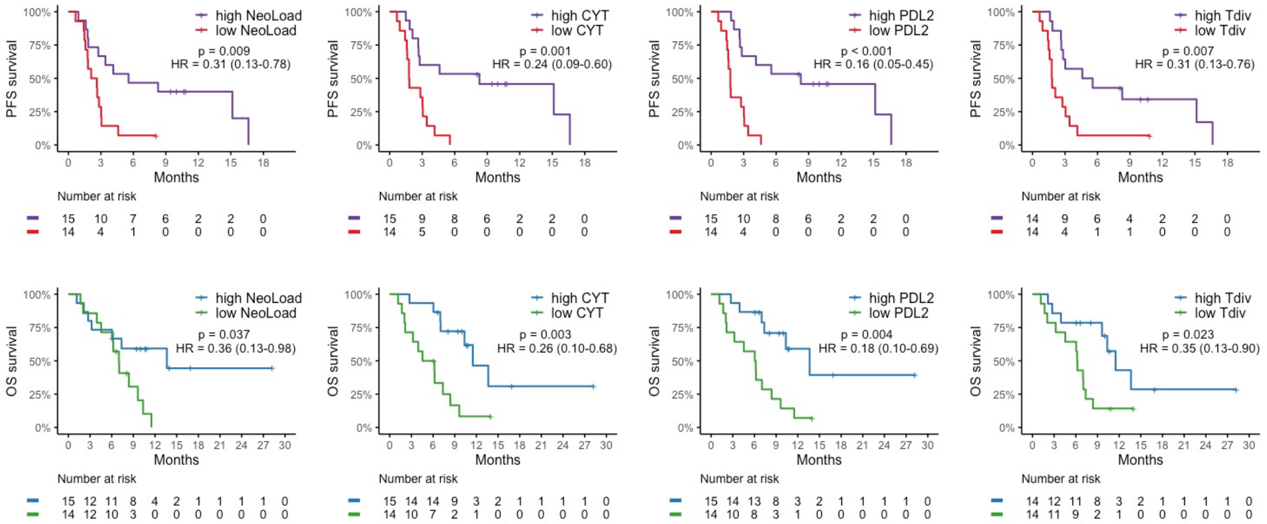

Supplementary Figure 3. Single biomarker survival analysis. Kaplan-Meyer curves for the single biomarkers. The groups got "high" and "low" are separated by the median for each biomarker. The single biomarkers include; predicted neoepitopes (NeoLoad), cytolytic activity (CYT), PD-L2 expression (PDL2), and T cell diversity (Tdiv). The hazard ratios, with confidence interval and the p-values, are calculated using cox-regression with survdiff log-rank test for p-values.

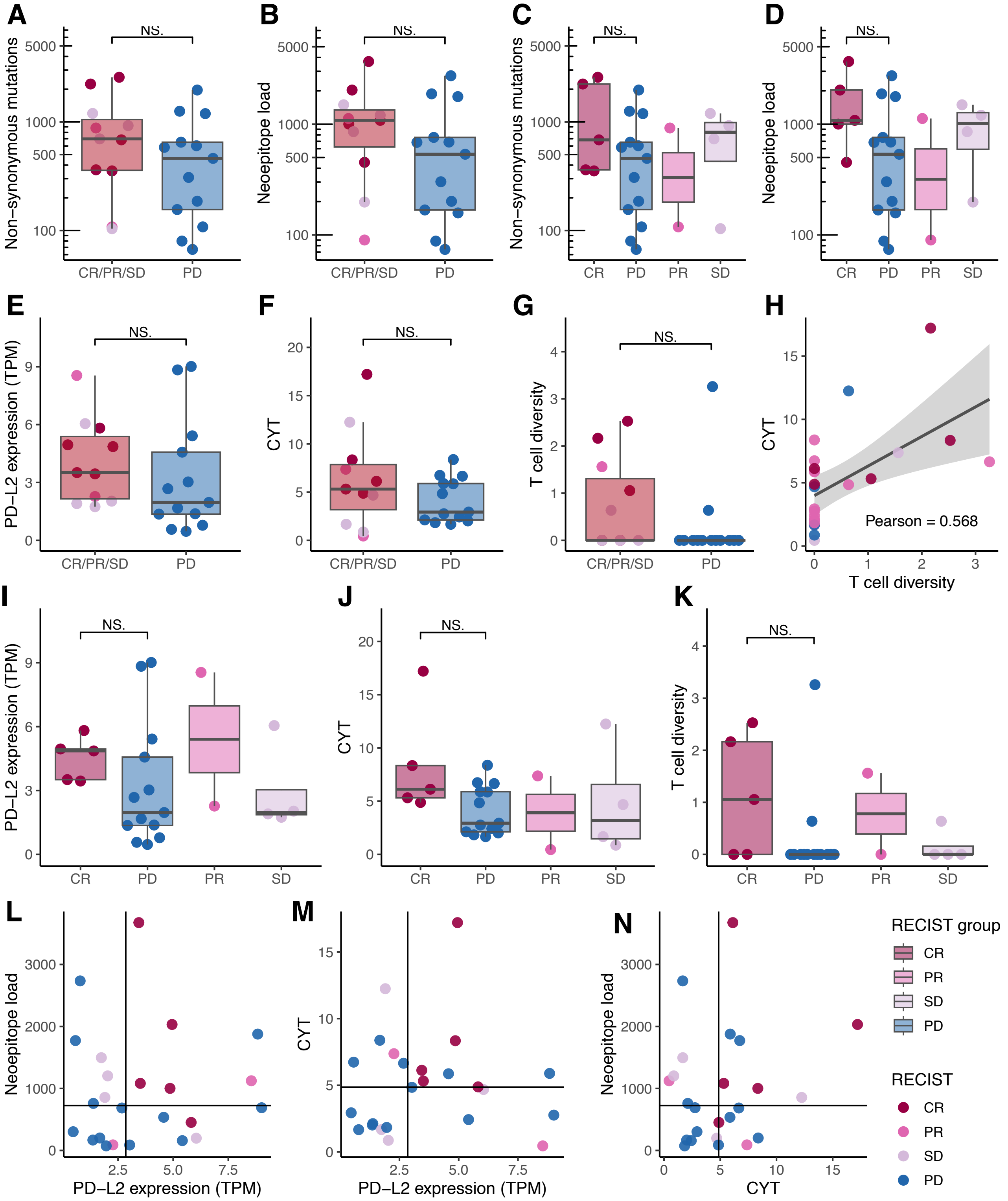

Supplementary Figure 4. Analysis from validation cohort. The analysis made from the validation cohort compared the suggested biomarker with RECIST criteria all tests comparing are performed with the Wilcox rank sum test and besides the correlations which were performed with person correlation. **A+B**) Comparing non-progressive and progressive from the RECIST group CR/PR/SD vs. SD. **A)** No significant differences were obtained by observing Tumor Mutational burden (TMB) (p-value = 0.213). **B)** Neither for the Neoepitope load (p-value = 0.119). **C+D)** Comparing

RECIST group individually and statistical differences between CR and PD were observed. **C)** For the TMB no significant difference were obtained (p-value = 0.143). **D)** Observing the neoepitope load resulted in a better separation but non-significant (p-value = 0.076). **E-G)** Comparing non-progressive and progressive patients. **E)** PD-L2 expression (TPM) (p-value = 0.150). **F)** Cytolytic activity (CYT) (p-value = 0.494). **G)** T cell diversity (p-value = 0.139). **H)** T cell diversity and CYT (person correlation = 0.568). **I-K)** Comparing RECIST criteria individually where statistic test comparing PD with CR. **I)** PD-L2 expression (p-value = 0.117). **J)** CYT (p-value = 0.076). **K)** T cell diversity (p-value = 0.081). **L-N)** Combination of some of the suggested biomarkers where the horizontal and vertical lines indicate the median. **L)** Predicted neoepitopes vs. PD-L2 expression. **M)** CYT vs. PD-L2 expression **N)** Neoepitope load vs. CYT.
